# Supplementary material for: Versatile kit of robust nanoshapes self-assembling from RNA and DNA modules
Source: Nat Commun. 2019 Feb 5;10:608. doi: 10.1038/s41467-019-08521-6 (PMC6363791; doi:10.1038/s41467-019-08521-6)
Supplement: Supplementary file 1 — Supplementary Information [file 41467_2019_8521_MOESM1_ESM.pdf]

# Supplementary Figures and Tables

# for

## Versatile kit of robust nanoshapes self-assembling from RNA and DNA modules

Monferrer *et al.*

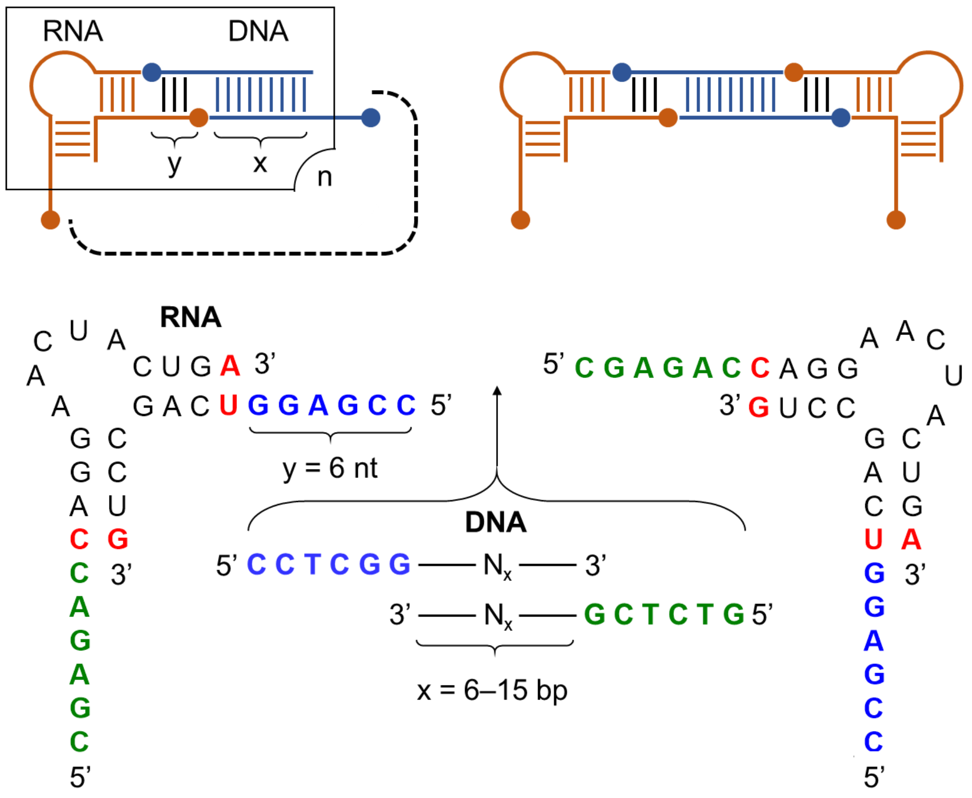


**Supplementary Figure 1. Design of RNA-DNA hybrid nanoshapes.** RNA corner modules are derived from the subdomain IIa internal loop of the hepatitis C virus (HCV) internal ribosome entry site (IRES), which was previously used to construct a self-assembling all-RNA nanosquare [1]. Nucleotides shown in color are changed from the module used in the all-RNA nanosquare. A series of DNA module inserts was designed with a base-paired region (x) varying from 6-15 base pairs (bp) (see Supplementary Fig. 2). RNA corner modules and DNA inserts each carry complementary 5' single-stranded overhangs (y) of 6 nucleotides (nt) which allow for self-assembly through base pairing recognition. Single-stranded overhangs within modules have distinct sequences (indicated by green and blue color) to prevent formation of homomeric assemblies.

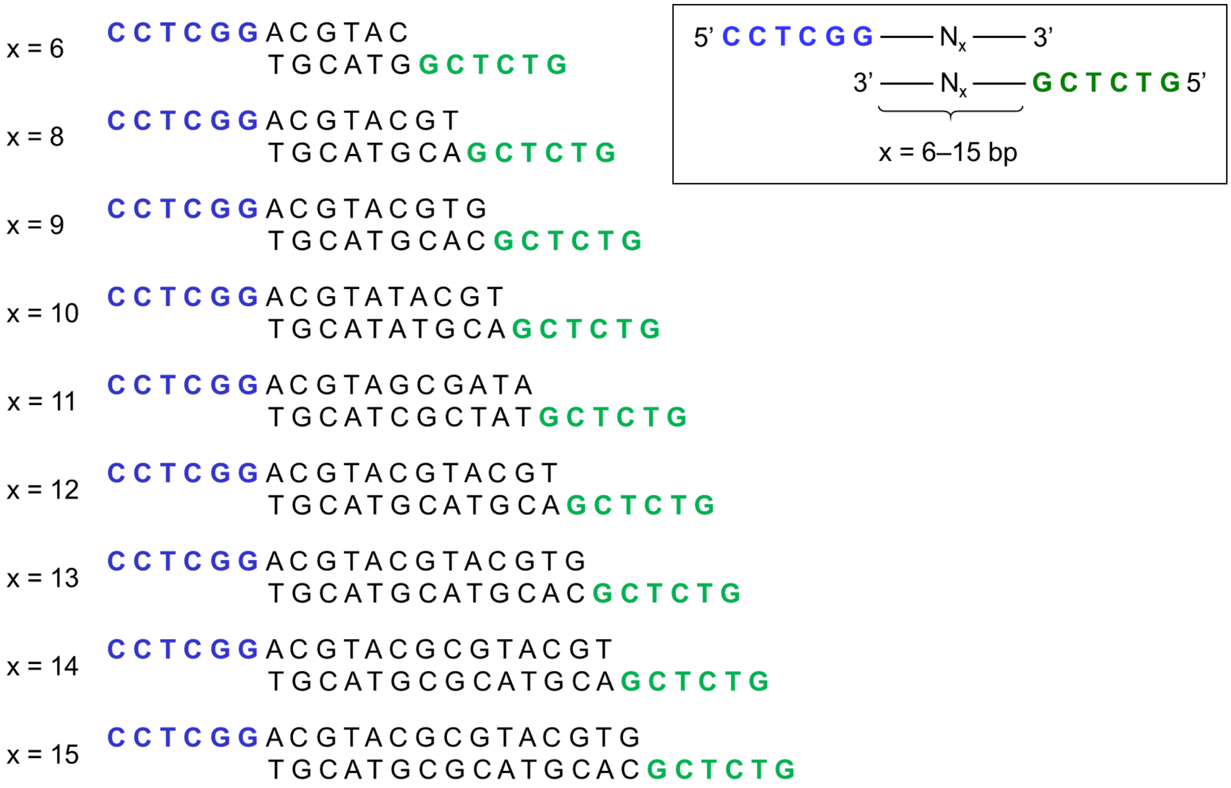


**Supplementary Figure 2. DNA modules for RNA-DNA hybrid nanoshapes.** (see also Supplementary Fig. 1) The DNA with 11 bp (DNA-11) gave the most stable RNA-DNA nanoshapes (RNA-DNA-11) which were used for all other experiments, except when indicated otherwise.

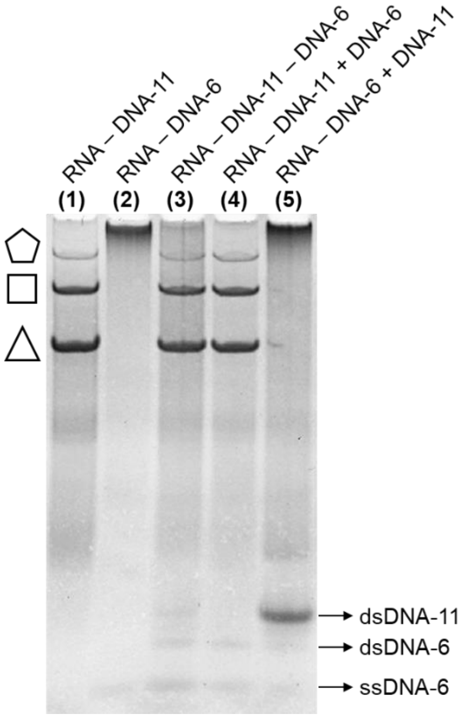


**Supplementary Figure 3. Kinetic stability of RNA-DNA hybrid nanoshapes.** Stability of RNA-DNA assemblies was tested at room temperature in the presence of competing DNA modules by native PAGE analysis. RNA-DNA-11 (lane 1) is the originally identified mixture of polygonal nanoshapes containing the RNA corner module and DNA inserts with 11 bp. RNA-DNA-6 (lane 2) is a mixture of the RNA corner module and DNA module with 6 bp which, after annealing, gave rise to high molecular weight oligomeric species that migrate only slowly. Annealing of the RNA corner module in the presence of a mixture of both modules, DNA-11 and DNA-6, the circularly closed RNA-DNA-11 nanoshapes form exclusively (RNA-DNA-11/DNA-6, lane 3), suggesting kinetic preference for assembling polygonal nanoshapes over oligomeric species. When pre-formed RNA-DNA-11 nanoshaphes are mixed at room temperature with DNA-6 module (RNA-DNA-11 + DNA-6, lane 4), or pre-formed RNA-DNA-6 oligomers with DNA-11 module (RNA-DNA-6 + DNA-11, lane 5), the originally formed assemblies each are stable and, at room temperature, do not interconvert by incorporation of the added DNA modules, demonstrating thermodynamic stability of both polygonal and oligomeric hybrid assemblies. Source data are provided as a Source Data file.

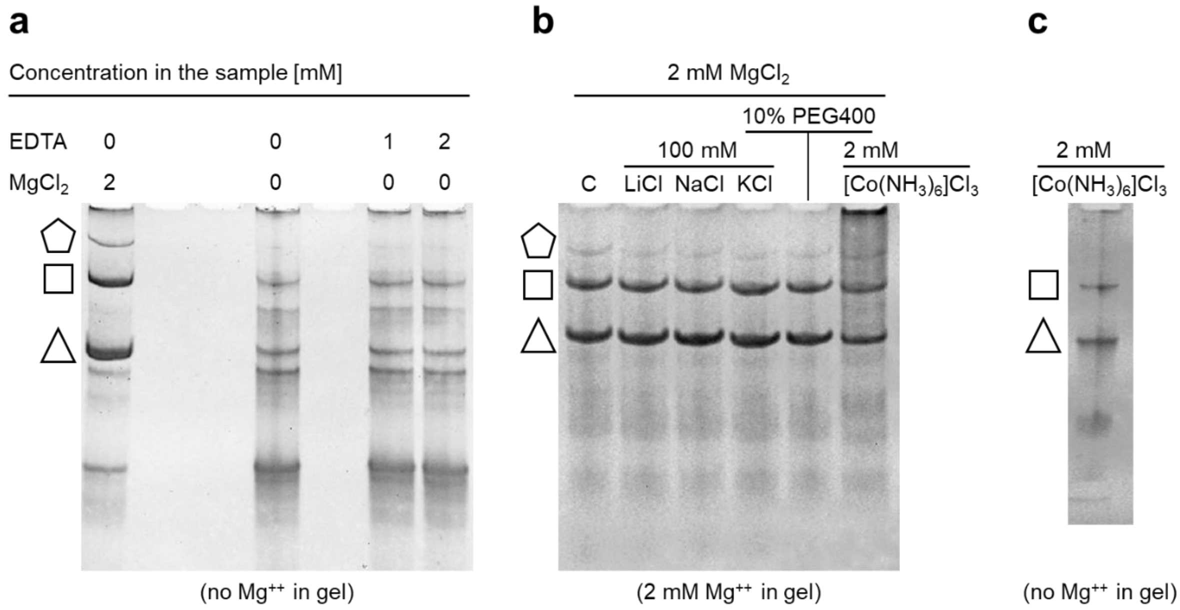


**Supplementary Figure 4. Ion requirement of RNA-DNA hybrid nanoshapes. a,** Native PAGE analysis of RNA-DNA-11 nanoshapes self-assembled in the absence and presence of magnesium ions. Formation of nanoshapes is observed even in the absence of magnesium, but to a much lower extent than in the presence of 2 mM magnesium. EDTA was used to remove potential magnesium contaminations in buffer. **b,** Monovalent salts and polyethylene glycol (average molecular mass 400, PEG400) did not affect formation of the nanoshapes in the presence of 2 mM magnesium, but 2 mM cobalt(III) hexammine promoted aggregation. Cobalt(III) hexammine ($Co(NH_3)_6^{3+}$) is known to interact with RNA preferably at sites otherwise occupied by magnesium ions. C is a control containing the mixture of nanoshapes assembled in the presence of 2 mM magnesium salt only (see Fig. 1d). **c,** Nanoshapes form in the presence of 2 mM cobalt(III) hexammine only, without magnesium ions present. Source data are provided as a Source Data file.

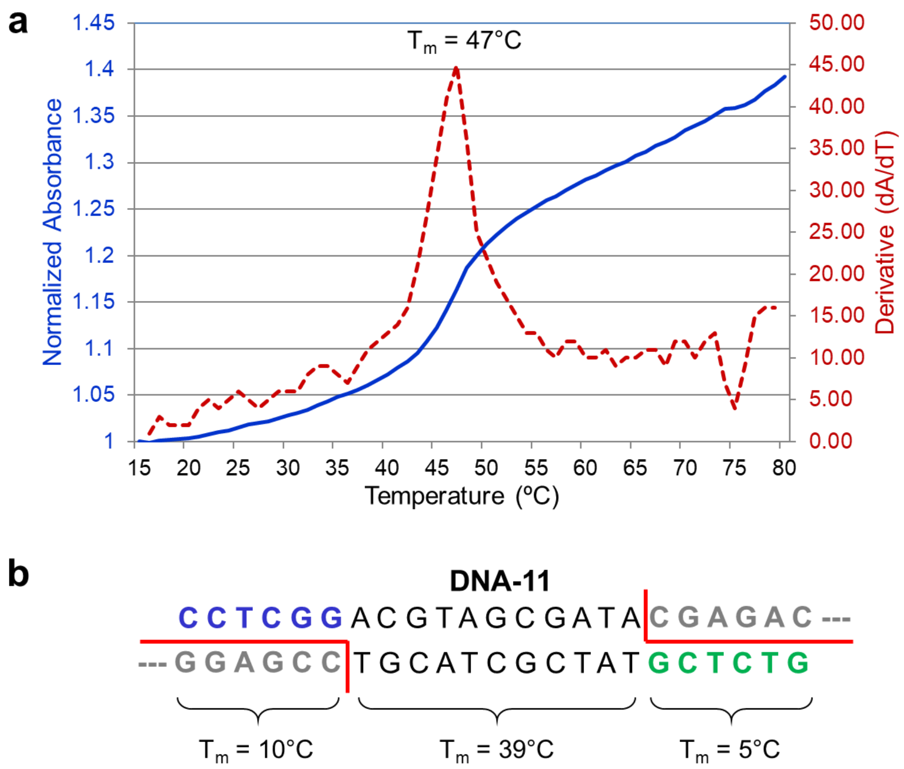


**Supplementary Figure 5. Thermal stability analysis of RNA-DNA hybrid nanoshapes. a,** UV absorption at 260 nm was monitored while heating a sample of RNA-DNA-11 nanoshapes in the presence of 2 mM magnesium salt. A single melting transition was observed at 47°C, suggesting that dissociation of the RNA and DNA modules occurs in concert with strand dissociation within the modules for all nanoshapes simultaneously. **b,** Predicted melting temperatures for the base-paired core of the DNA-11 insert and the overhanging sequences that hybridize to the RNA corner module. Prediction is based on the Nearest-Neighbor method [2] adjusted for 2 mM magnesium ion concentration [3].

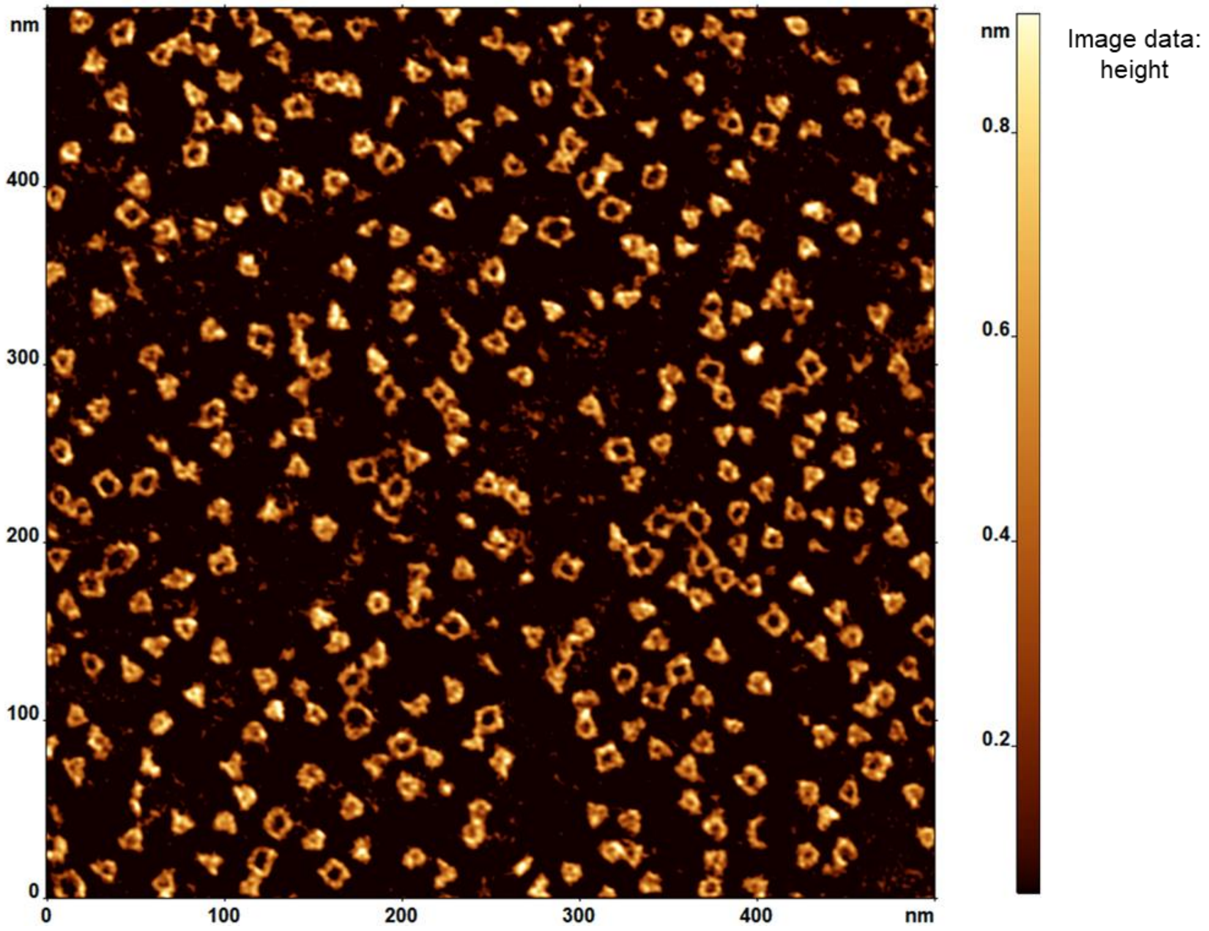


**Supplementary Figure 6. AFM imaging field of RNA-DNA hybrid nanoshapes.** Imaging was performed on the mixture of polygonal RNA-DNA-11 nanoshapes identified by PAGE analysis screening (see Fig. 1d, 2b).

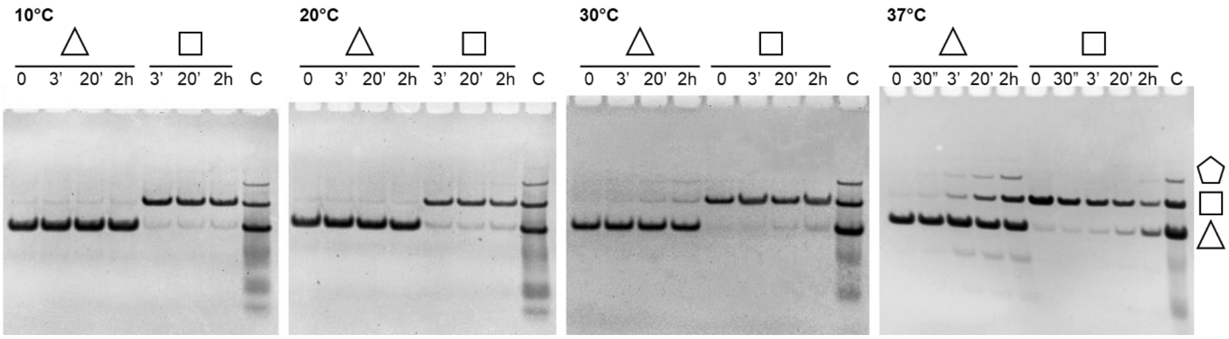


**Supplementary Figure 7. Thermal stability and re-equilibration of RNA-DNA hybrid nanoshapes.** Temperature-dependent stability over time of purified RNA-DNA-11 hybrid nanoshapes analyzed by native PAGE in the presence of 2 mM magnesium salt. Discrete triangles and squares are stable at 20°C but slowly re-equilibrate to a mixture of nanoshapes at 37°C. C is a control containing the mixture of polygonal nanoshapes before separation of discrete structures (see Fig. 1d). Source data are provided as a Source Data file.

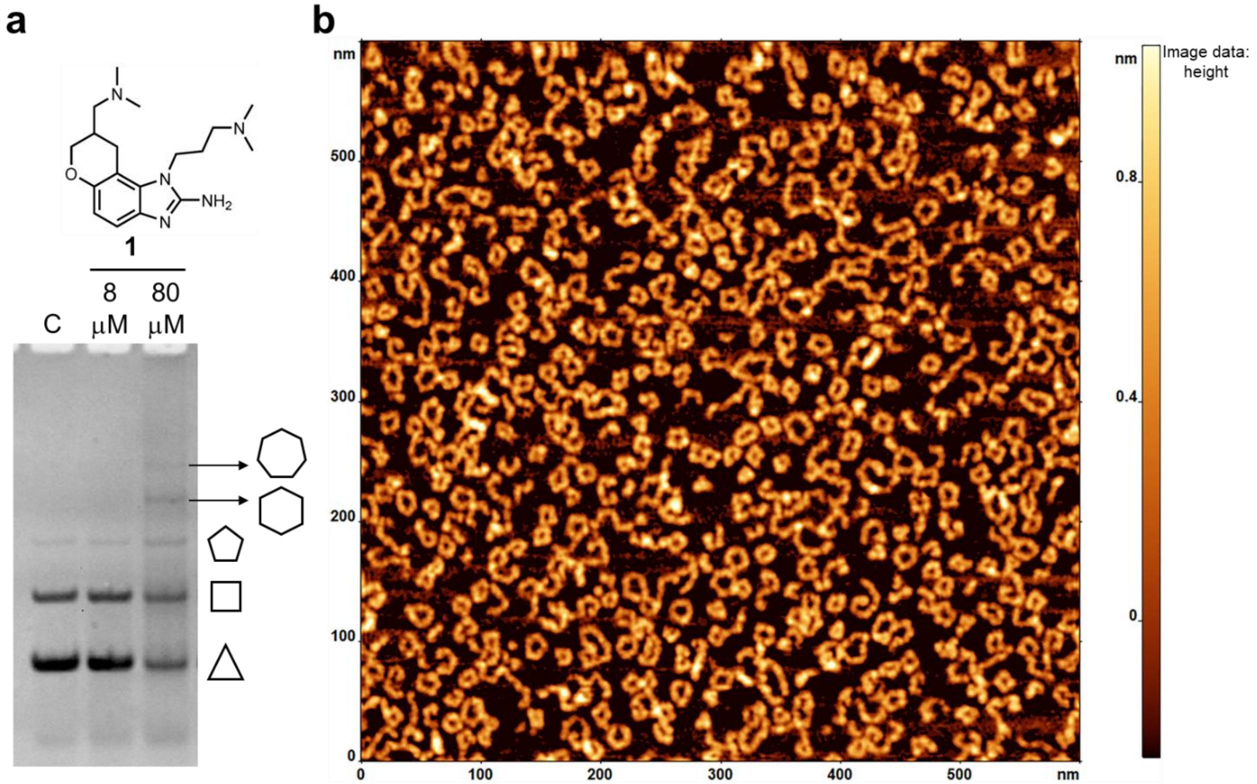


**Supplementary Figure 8. Small molecule control of RNA-DNA hybrid nanoshapes. a,** The HCV IRES subdomain IIa internal loop used as RNA corner modules for the nanoshape design contains a binding site for 2-amino-benzimidazole viral translation inhibitors which capture the RNA in an extended conformation [4]. Formation of larger ring structures, including hexagons and heptagons, is observed by native PAGE in the presence of a benzimidazole ligand which increases the availability of extended RNA modules in equilibrium during the formation of polygonal nanoshapes. C is a control containing the mixture of polygonal nanoshapes (see Fig. 1d). **b,** AFM imaging field of RNA-DNA hybrid nanoshape mixture formed in the presence of 80 µM benzimidazole ligand **1**. Larger polygons are enriched (compare Supplementary Fig. 6). Source data are provided as a Source Data file.

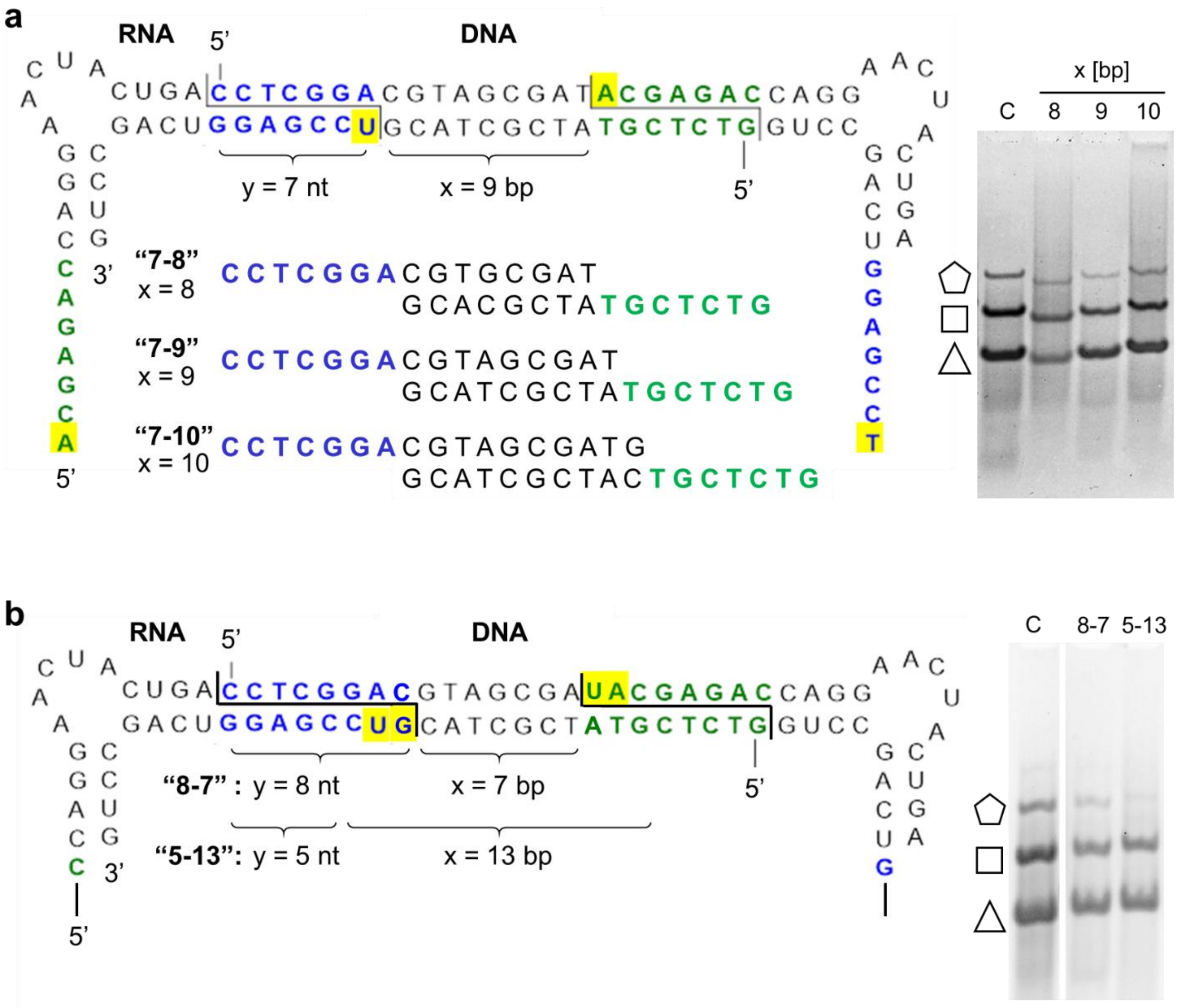


**Supplementary Figure 9. Design of RNA-DNA hybrid nanoshapes with increased stability and variation in overhang lengths. a,** The original design shown in Supplementary Fig. 1 was modified by extending the single-stranded overhangs in RNA and DNA modules by one nucleotide while removing one to three base pairs from the DNA insert to compensate for the length increase of the RNA-DNA hybrid region (7-8, 7-9, 7-10 constructs). Native PAGE analysis shows increased stability of the resulting nanoshapes which tolerated a range of DNA inserts (compare Fig. 1d). C is a control containing the mixture of original design RNA-DNA-11 polygonal nanoshapes. **b,** Both, further extension of the single-stranded overhangs to 8 nt (8-7 construct) or shortening to 5 nt (5-13 construct) in concert with adjustment of the double-stranded region to 7 or 13 bp, respectively, was compatible with the formation of the RNA-DNA nanoshapes as demonstrated by native PAGE analysis. Source data are provided as a Source Data file.

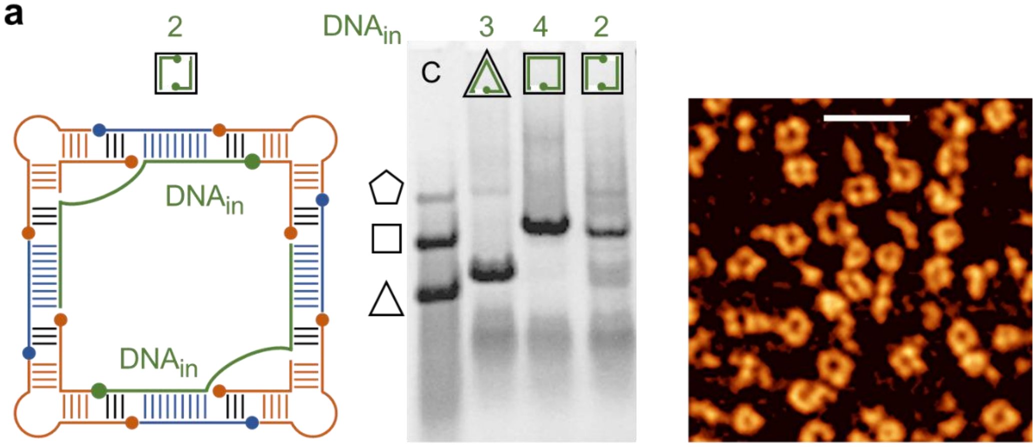


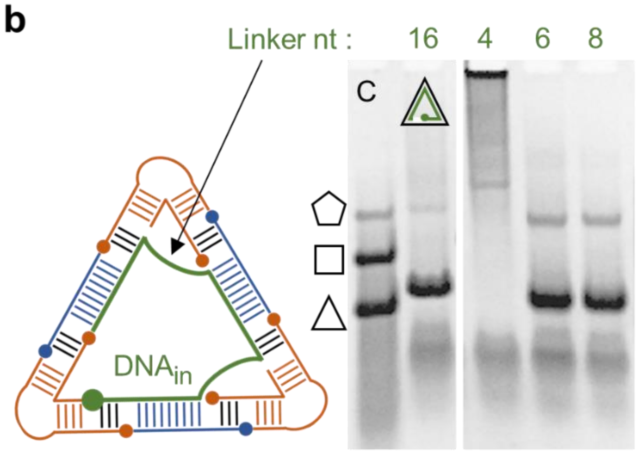


**Supplementary Figure 10. Homogenous RNA-DNA hybrid nanoshapes containing DNA guide strand modules with two hybridization sites and variations in linker length. a)** A DNA guide strand (green) with two hybridization sites, spaced by a linker sequence, directs formation of nanosquares by inclusion of two guide strands as revealed by native PAGE and AFM topography imaging. Scale bar in the AFM image is 50 nm. **b)** Variation of the linker length between hybridization sites in DNA guide strands was tested in homogenous nanotriangles. Native PAGE analysis revealed that linkers containing 6, 8 or 16 nt permitted formation of clean nanoshapes while a short linker sequence of 4 nt was not tolerated. Source data are provided as a Source Data file.

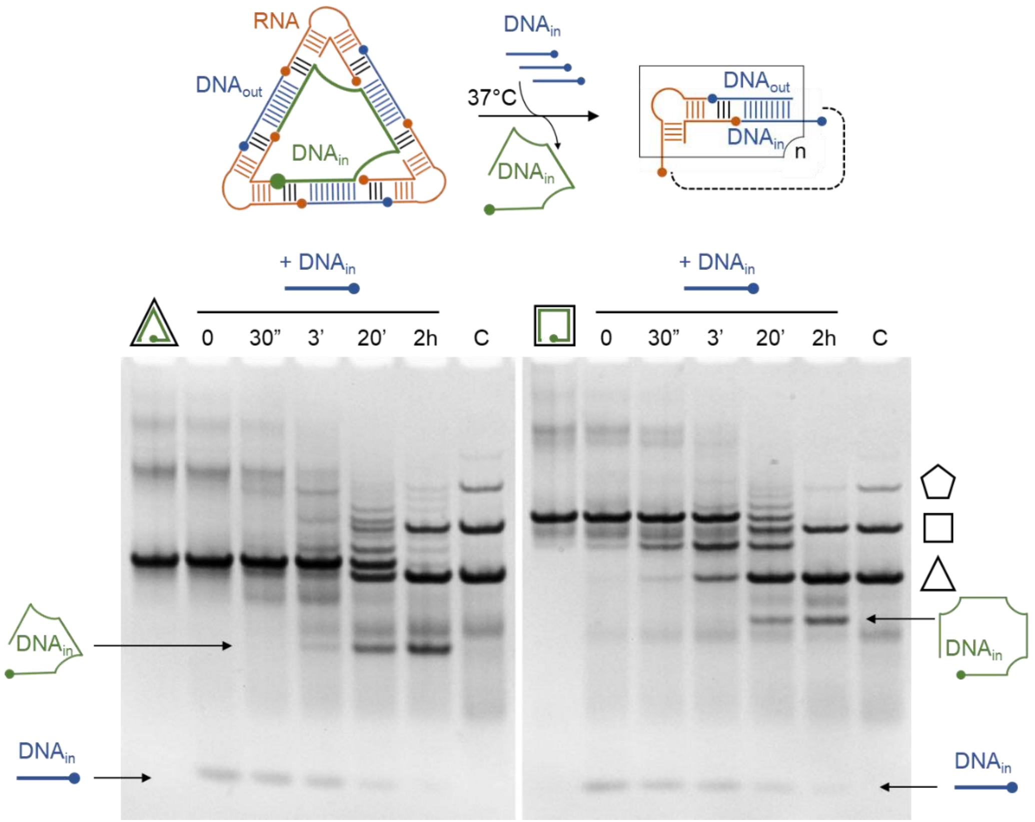


**Supplementary Figure 11. Controlled conversion of homogenous RNA-DNA hybrid nanoshapes.** Addition of short strands from the original DNA insert modules (see Supplementary Fig. 1, 2) to homogenous RNA-DNA nanoshapes, which contain a single $DNA_{in}$ guide with multiple hybridization sites, leads to slow conversion of the homogenous nanoshapes and to a mixture of RNA-DNA-11 polygonal nanoshapes at 37°C. The re-equilibration proceeded through intermediate assemblies in which the long DNA guide may have been partially displaced by the competing short DNA-11 strand (visible at the 20 minute time point). C is a control containing the mixture of nanoshapes (see Fig. 1d). Source data are provided as a Source Data file.

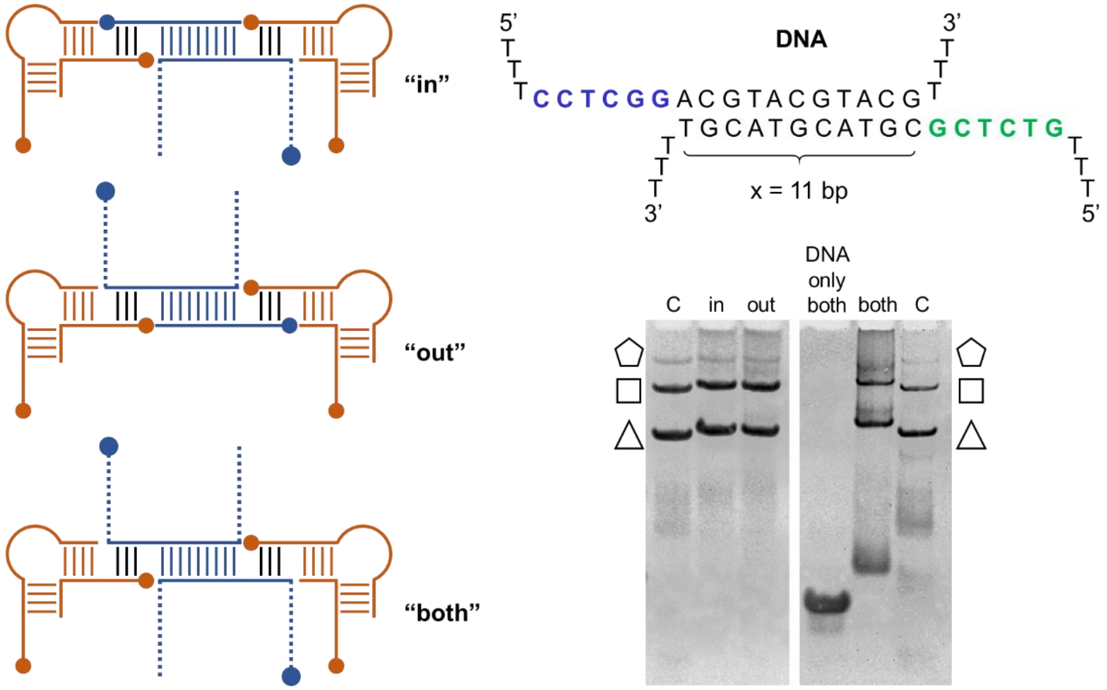


**Supplementary Figure 12. DNA modules with ssDNA overhangs in RNA-DNA hybrid nanoshapes.** The simple double-stranded DNA module inserts can be modified or replaced to increase the structural and functional diversity of RNA-DNA nanoshapes. Native PAGE analysis shows that extended DNA strands are tolerated in the RNA-DNA-11 nanoshapes, which will allow addition of interaction sites for nucleic acids and proteins as well as conjugation by covalent modification. C is a control containing the mixture of nanoshapes (see Fig. 1d). Source data are provided as a Source Data file.

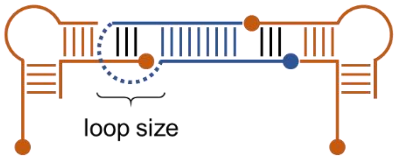


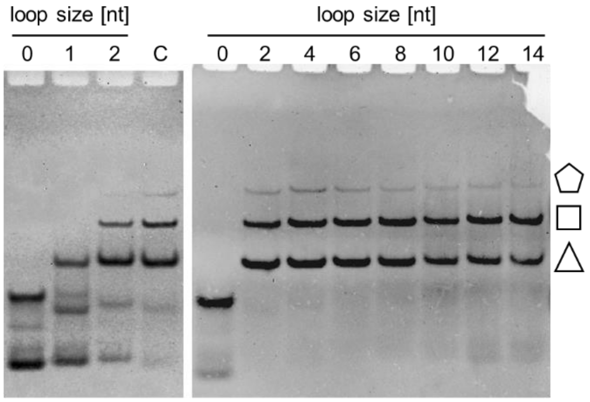


loop size [nt]

0 5' **GTCTCG**TATCGCTACGT**CCTCGG**ACGTAGCGATA 3'

1 5' **GTCTCG**TATCGCTACGT**T****CCTCGG**ACGTAGCGATA 3'

2 5' **GTCTCG**TATCGCTACGT**TT****CCTCGG**ACGTAGCGATA 3'

4 5' **GTCTCG**TATCGCTACGT**TTTT****CCTCGG**ACGTAGCGATA 3'

6 5' **GTCTCG**TATCGCTACGT**TTTTTT****CCTCGG**ACGTAGCGATA 3'

8 5' **GTCTCG**TATCGCTACGT**TTTCTTTT****CCTCGG**ACGTAGCGATA 3'

10 5' **GTCTCG**TATCGCTACGT**TTTCTCTTTT****CCTCGG**ACGTAGCGATA 3'

12 5' **GTCTCG**TATCGCTACGT**TTTCTCTCTTTT****CCTCGG**ACGTAGCGATA 3'

14 5' **GTCTCG**TATCGCTACGT**TTTCTCTCTCTTTT****CCTCGG**ACGTAGCGATA 3'

**Supplementary Figure 13. Hairpin DNA modules in RNA-DNA hybrid nanoshapes.** Double-stranded DNA module inserts in the RNA-DNA-11 nanoshapes can be connected by a hairpin in unimolecular DNA inserts. Loop nucleotides are underlined. Native PAGE analysis shows that a variety of loop sizes, and at the lower limit comprising only 2 nt, are sufficient to furnish stable nanoshapes. C is a control containing the mixture of nanoshapes (see Fig. 1d). Source data are provided as a Source Data file.

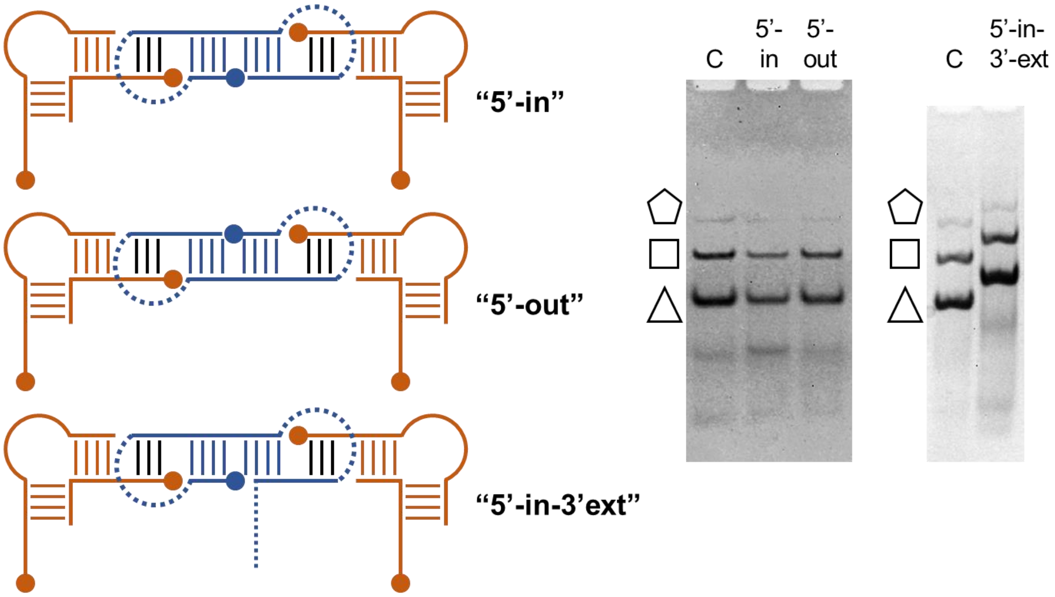


**“5’-in”** 5’ TACGT**TTCCTCGG**ACGTAGCGATA**TTGTCTCG**TATCGC 3’

**“5’-out”** 5’ GCGATA**TTGTCTCG**TATCGCTACGT**TTCCTCGG**ACGTA 3’

**“5’-in-3’ext”** 5’ CTACGT**TTTTCCTCGG**ACGTAGCGATA**TTTTGTCTCG**TATCG**TTTCTCTCTTTT** 3’

**Supplementary Figure 14. Circularly permutated hairpin DNA modules in RNA-DNA hybrid nanoshapes.** Circular permutations of DNA inserts containing hairpins with as few as 2 nt (see Supplementary Fig. 13) can be used to form stable RNA-DNA-11 nanoshapes, as demonstrated by native PAGE. The permutated termini may reside in either strand of the DNA insert (5’-in or 5’-out). Terminal single stranded overhangs in the circularly permutated hairpin DNA modules are tolerated as well (5’-in-3’-ext). C is a control containing the mixture of nanoshapes (see Fig. 1d). Source data are provided as a Source Data file.

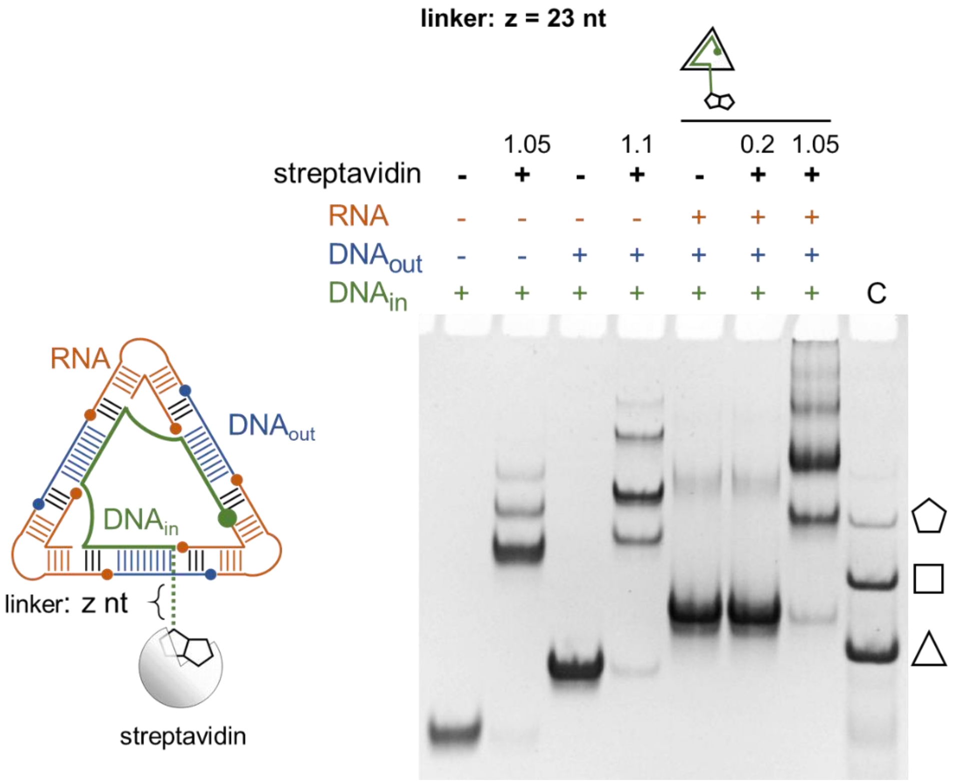


**Supplementary Figure 15. Streptavidin binding to homogenous RNA-DNA hybrid nanotriangles.** Streptavidin binding to homogenous nanotriangles with a DNA guide strand (green) that contains three hybridization sites and a 3' single strand extension conjugated with biotin, z = 13 nt (see Fig. 6b). Native PAGE analysis demonstrates formation of nanotriangle complexes with streptavidin. Streptavidin contains 4 binding sites for biotin. At a stoichiometric ratio of protein to nucleic acid, 3 discrete complexes are observed for the DNA hybrid ($DNA_{in}$-$DNA_{out}$) and the nanotriangle, presumably corresponding to 1-4 nucleic acid species bound to one streptavidin. Similar results were obtained when extending the linker to z = 23 nt. Source data are provided as a Source Data file.

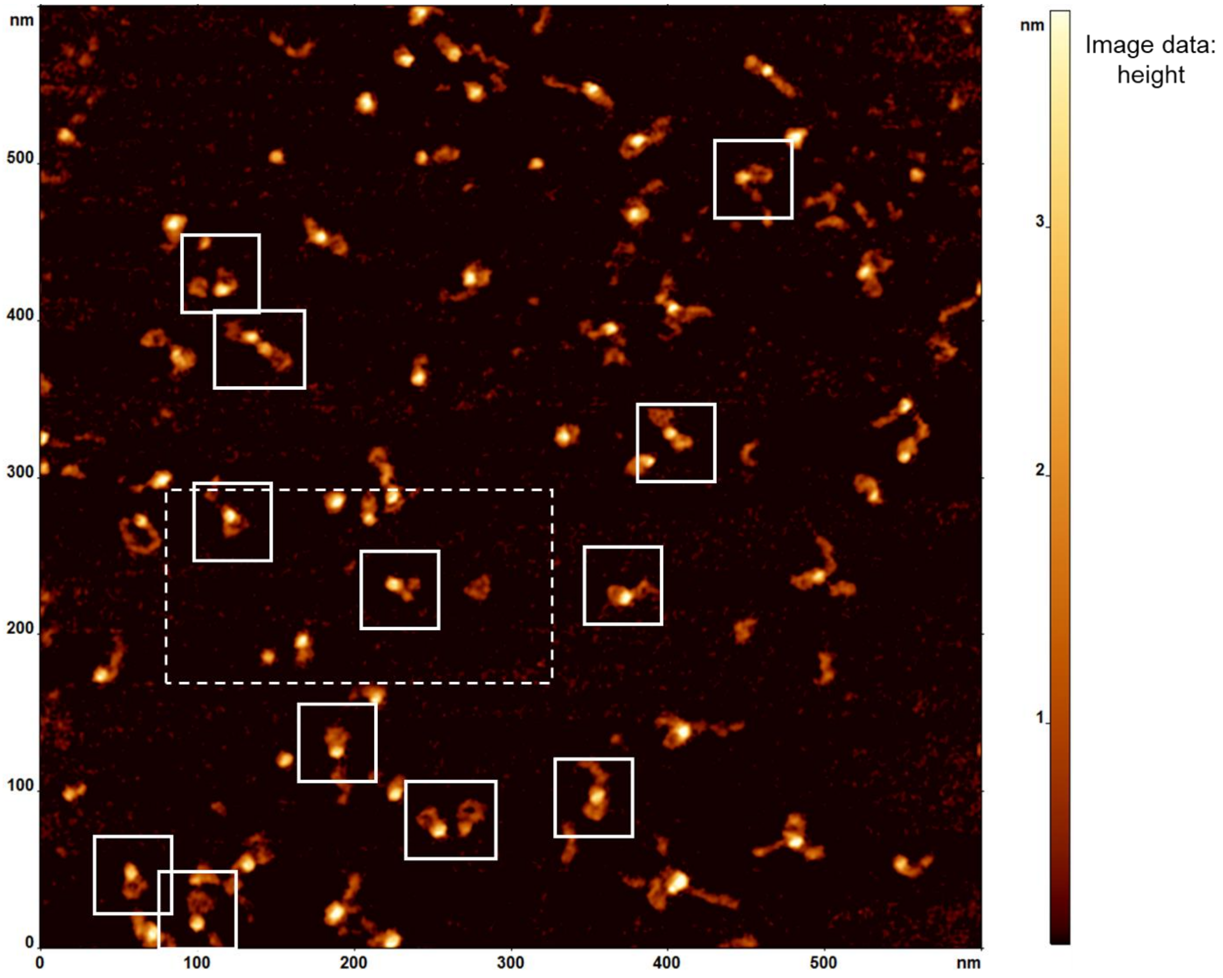


**Supplementary Figure 16. AFM topography image of streptavidin-bound homogenous RNA-DNA hybrid nanotriangles.** Imaging was performed on a sample of streptavidin-bound homogenous nanotriangles with a DNA strand that contains three hybridization sites and a 3' single strand extension conjugated with biotin (see Fig. 6b, Supplementary Fig. 15). Solid boxes indicate complexes showing a clearly visible nanotriangle with an attached streptavidin. Other visible streptavidin complexes may represent complexes with several nanotriangles bound to a single streptavidin or complexes that present the nucleic acid components in a distorted or disrupted state. The dashed box indicates the area used to illustrate height analysis of nanotriangle complexes in Supplementary Fig. 17.

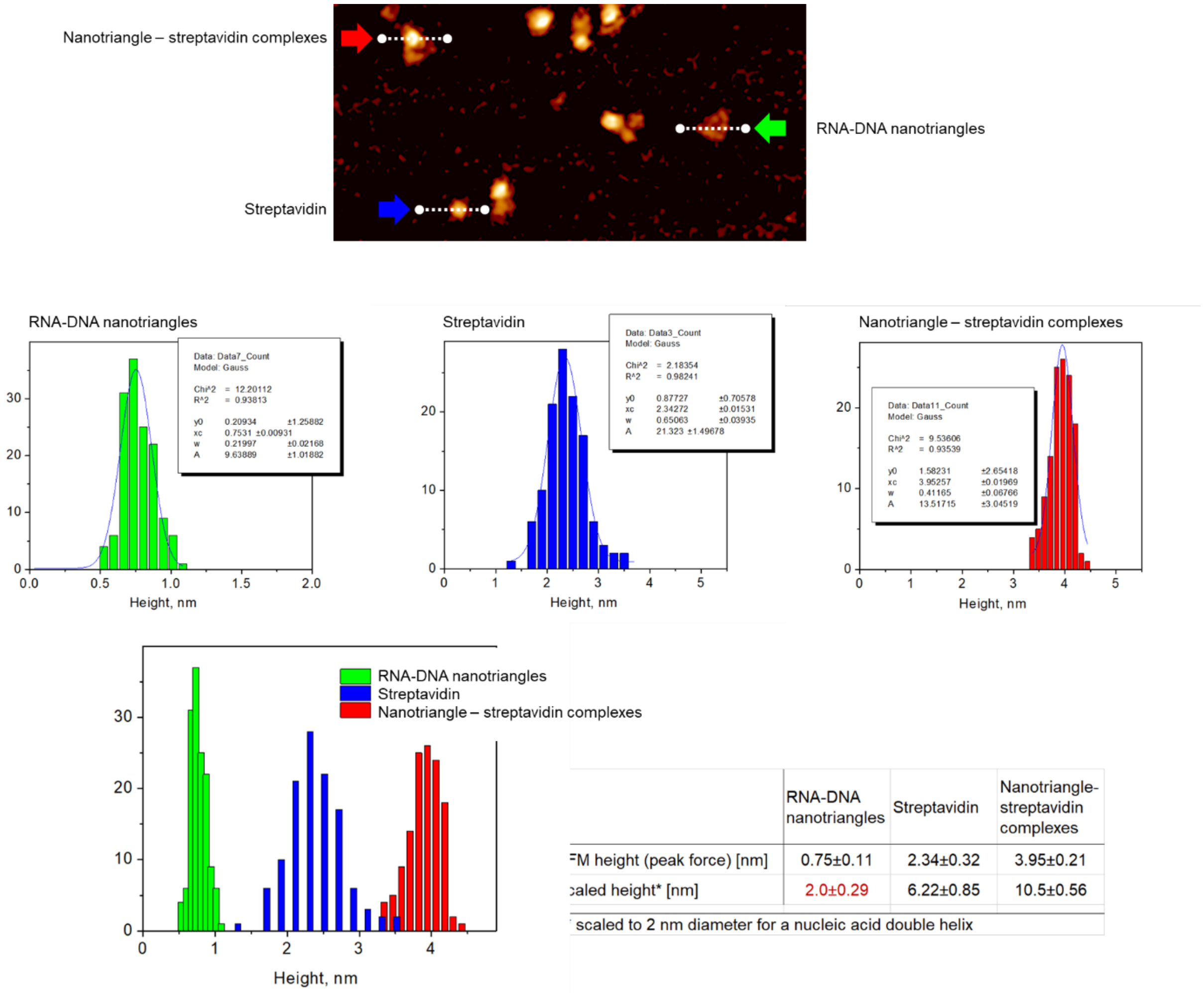


| | RNA-DNA nanotriangles | Streptavidin | Nanotriangle-streptavidin complexes |
|---|---|---|---|
| FM height (peak force) [nm] | 0.75±0.11 | 2.34±0.32 | 3.95±0.21 |
| caled height* [nm] | 2.0±0.29 | 6.22±0.85 | 10.5±0.56 |

' scaled to 2 nm diameter for a nucleic acid double helix

**Supplementary Figure 17. AFM height analysis of streptavidin-bound homogenous RNA-DNA hybrid nanotriangles.** AFM height analysis in peak force mode was used on a sample of streptavidin-bound homogenous nanotriangles with a DNA strand that contains three hybridization sites and a 3' single strand extension conjugated with biotin (see Fig. 6b, Supplementary Figs 15, 16). Height was measured of particles identified as nucleic acid nanotriangles (green), free streptavidin protein (blue) and across the streptavidin component of nanotriangle-streptavidin complexes (red). Gauss approximation was used to fit histograms and calculate particle height from peak force mode. The table shows peak heights from the Gauss analysis and heights scaled assuming a nucleic acid helix diameter of 2 nm.

**Table 1.** RNA oligonucleotides sequences for corner units in RNA-DNA hybrid nanoshapes.

| | |
|---|---|
| RNA corner inner strand | 5' – rCrCrG rArGrG rUrCrA rGrCrC rUrG – 3' |
| RNA corner outer strand | 5' – rCrGrA rGrArC rCrArG rGrArA rCrUrA rCrUrG rA – 3' |
| Antisense to corner inner strand (Fig. 3b) | 5' – rGrCrA rGrArC rCrArG rGrCrU rGrA – 3' |

**Table 2.** DNA oligonucleotide sequences for inserts in RNA-DNA hybrid nanoshapes.

| | |
|---|---|
| DNA-6-in* | 5’ – GTC TCG GTA CGT – 3’ |
| DNA-6-out | 5’ – CCT CGG ACG TAC – 3’ |
| DNA-8-in | 5’ – GTC TCG ACG TAC GT – 3’ |
| DNA-8-out | 5’ – CCT CGG ACG TAC GT – 3’ |
| DNA-9-in | 5’ – GTC TCG CAC GTA CGT – 3’ |
| DNA-9-out | 5’ – CCT CGG ACG TAC GTG – 3’ |
| DNA-10-in | 5’ – GTC TCG ACG TAT ACG T – 3’ |
| DNA-10-out | 5’ – CCT CGG ACG TAT ACG T – 3’ |
| DNA-11-in | 5’ – GTC TCG TAT CGC TAC GT – 3’ |
| DNA-11-out | 5’ – CCT CGG ACG TAG CGA TA – 3’ |
| DNA-12-in | 5’ – GTC TCG ACG TAC GTA CGT – 3’ |
| DNA-12-out | 5’ – CCT CGG ACG TAC GTA CGT – 3’ |
| DNA-13-in | 5’ – GTC TCG CAC GTA CGT ACG T – 3’ |
| DNA-13-out | 5’ – CCT CGG ACG TAC GTA CGT G – 3’ |
| DNA-14-in | 5’ – GTC TCG ACG TAC GCG TAC GT – 3’ |
| DNA-14-out | 5’ – CCT CGG ACG TAC GCG TAC GT – 3’ |
| DNA-15-in | 5’ – GTC TCG CAC GTA CGC GTA CGT – 3’ |
| DNA-15-out | 5’ – CCT CGG ACG TAC GCG TAC GTG – 3’ |
| DNA-21-in | 5’ – GTC TCG TGC ACT CGA TTA TCG CTA CGT – 3’ |
| DNA-21-out | 5’ – CCT CGG ACG TAG CGA TAA TCG AGT GCA – 3’ |
| DNA-31-in | 5’ – GTC TCG CGA ACA GTT CTG CAC TCG ATT ATC GCT ACG T – 3’ |
| DNA-31-out | 5’ – CCT CGG ACG TAG CGA TAA TCG AGT GCA GAA CTG TTC G – 3’ |
| DNA-41-in | 5’ – GTC TCG CGT CGA CTT ACG AAC AGT TCT GCA CTC GAT TAT CGC TAC GT – 3’ |
| DNA-41-out | 5’ – CCT CGG ACG TAG CGA TAA TCG AGT GCA GAA CTG TTC GTA AGT CGA CG – 3’ |
| DNA-50-in | 5’ – GTC TCG GAG CCA TAT CGT CGA CTA CGA TGA CTC ACT GCA CTC GAT TAT CGC TAC GT – 3’ |
| DNA-50-out | 5’ – CCT CGG ACG TAG CGA TAA TCG AGT GCA GTG AGT CAT CGT AGT CGA CGA TAT GGC TC – 3’ |
| DNA-51-in | 5’ – GTC TCG GGA GCC ATA TCG TCG ACT TAC GAA CAG TTC TGC ACT CGA TTA TCG CTA CGT – 3’ |
| DNA-51-out | 5’ – CCT CGG ACG TAG CGA TAA TCG AGT GCA GAA CTG TTC GTA AGT CGA CGA TAT GGC TCC – 3’ |

(* Numbers indicate the number of base pairs x in the DNA insert.)

**Table 3.** RNA oligonucleotides sequences for corner units in RNA-DNA hybrid nanoshapes with variation in overhang length (Supplementary Fig. 9).

| | |
|---|---|
| RNA corner 7-8/9/10 inner strand | 5' – rUrCrC rGrArG rGrUrC rArGrC rCrUrG – 3' |
| RNA corner 7-8/9/10 outer strand | 5' – rArCrG rArGrA rCrCrA rGrGrA rArCrU rArCrU rGrA – 3' |
| RNA corner 5-13 inner strand | 5' – rCrGrA rGrGr rUrCr rArGrC rCrUrG – 3' |
| RNA corner 5-13 outer strand | 5' – rGrArG rArCr rCrArG rGrArA rCrUrA rCrUrG rA – 3' |
| RNA corner 8-7 inner strand | 5' – rGrUrC rCrGrA rGrGrU rCrArG rCrCrU rG – 3' |
| RNA corner 8-7 outer strand | 5' – rUrArC rGrArG rArCrC rArGrG rArArC rUrArC rUrGrA – 3' |

**Table 4.** DNA oligonucleotide sequences for inserts in RNA-DNA hybrid nanoshapes with variation in overhang length (Supplementary Fig. 9).

| | |
|---|---|
| $DNA_7$-8-in* | 5' – GTC TCG TAT CGC ACG – 3' |
| $DNA_7$-8-out | 5' – CCT CGG ACG TGC GAT – 3' |
| $DNA_7$-9-in | 5' – GTC TCG TAT CGC TAC G – 3' |
| $DNA_7$-9-out | 5' – CCT CGG ACG TAG CGA T – 3' |
| $DNA_7$-10-in | 5' – GTC TCG TCA TCG CTA CG – 3' |
| $DNA_7$-10-out | 5' – CCT CGG ACG TAG CGA TG – 3' |
| $DNA_5$-13-in | 5' – GTC TCG TAT CGC TAC GTC – 3' |
| $DNA_5$-13-out | 5' – CCT CGG ACG TAG CGA TAC – 3' |
| $DNA_8$-7-in | 5' – GTC TCG TAT CGC TAC – 3' |
| $DNA_8$-7-out | 5' – CCT CGG ACG TAG CGA – 3' |

(* The index indicates the length of the overhang; the following number indicates the number of base pairs x in the DNA insert. For example, *DNA7-8-in* has an overhang of 7 nucleotides and forms 8 base pairs with *DNA7-8-out*.)

**Table 5.** DNA oligonucleotide sequences for inner guide strands in homogenous RNA-DNA hybrid nanoshapes (Fig. 5, Supplementary Figs 10, 11).

| | |
|---|---|
| DNA$_{guide}$-2-in* | 5' –**GTC TCG TAT CGC TAC GT**T TTC TCT CTC TCT TTT **GTC TCG TAT CGC TAC GT** – 3' |
| DNA$_{guide}$-3-in* (16 nt linker between hybridization sites) | 5' – TTT **GTC TCG TAT CGC TAC GT**T TTC TCT CTC TCT TTT **GTC TCG TAT CGC TAC GT**T TTC TCT CTC TCT TTT **GTC TCG TAT CGC TAC GT**T TT – 3' |
| DNA$_{guide}$-3-in* (4 nt linker between hybridization sites) | 5' – TTT **GTC TCG TAT CGC TAC GT**T TTT **GTC TCG TAT CGC TAC GT**T TTT **GTC TCG TAT CGC TAC GT**T TT – 3' |
| DNA$_{guide}$-3-in* (6 nt linker between hybridization sites) | 5' – TTT **GTC TCG TAT CGC TAC GT**T TCC TT**G TCT CGT ATC GCT ACG T**TT CCT T**GT CTC GTA TCC TAC GT**T TT – 3' |
| DNA$_{guide}$-3-in* (8 nt linker between hybridization sites) | 5' – TTT **GTC TCG TAT CGC TAC GT**T TCC TTC C**GT CTC GTA TCG CTA CGT** TTC CTT CC**G TCT CGT ATC GCT ACG T**TT T – 3' |
| DNA$_{guide}$-4-in* | 5' – TTT **GTC TCG TAT CGC TAC GT**T TTC TCT CTC TCT TTT **GTC TCG TAT CGC TAC GT**T TTC TCT CTC TCT TTT **GTC TCG TAT CGC TAC GT**T TTC TCT CTC TCT TTT **GTC TCG TAT CGC TAC GT**T TT – 3' |
| DNA$_{guide}$-5-in* | 5' – TTT **GTC TCG TAT CGC TAC GT**T TTC TCT CTC TCT TTT **GTC TCG TAT CGC TAC GT**T TTC TCT CTC TCT TTT **GTC TCG TAT CGC TAC GT**T TTC TCT CTC TCT TTT **GTC TCG TAT CGC TAC GT**T TTC TCT CTC TCT TTT GTC TCG TAT CGC TAC GTT TT – 3' |

(* Hybridization sites in the DNA guide are underlined.)

**Table 6.** DNA oligonucleotide sequences for topologically diverse inserts in functionalized RNA-DNA hybrid nanoshapes (Fig. 6a, Supplementary Figs 12, 13, 14).

| | |
|---|---|
| Supp. Fig. 12: overhangs | |
| DNA$_{\text{overhangs}}$-in* | 5’ – **TTT** GTC TCG TAT CGC TAC GT**T TT** – 3’ |
| DNA$_{\text{overhangs}}$-out* | 5’ – **TTT** CCT CGG ACG TAG CGA TA**T TT** – 3’ |
| Supp. Fig. 13: hairpin | |
| DNA$_{\text{hairpin}}$-0nt-loop | 5’ – GTC TCG TAT CGC TAC GTC CTC GGA CGT AGC GAT A – 3’ |
| DNA$_{\text{hairpin}}$-1nt-loop* | 5’ – GTC TCG TAT CGC TAC GT**T** CCT CGG ACG TAG CGA TA – 3’ |
| DNA$_{\text{hairpin}}$-2nt-loop* | 5’ – GTC TCG TAT CGC TAC GT**T T**CC TCG GAC GTA GCG ATA – 3’ |
| DNA$_{\text{hairpin}}$-4nt-loop* | 5’ – GTC TCG TAT CGC TAC GT**T TTT** CCT CGG ACG TAG CGA TA – 3’ |
| DNA$_{\text{hairpin}}$-6nt-loop* | 5’ – GTC TCG TAT CGC TAC GT**T TTT TT**C CTC GGA CGT AGC GAT A – 3’ |
| DNA$_{\text{hairpin}}$-8nt-loop* | 5’ – GTC TCG TAT CGC TAC GT**T TTC TTT T**CC TCG GAC GTA GCG ATA – 3’ |
| DNA$_{\text{hairpin}}$-10nt-loop* | 5’ – GTC TCG TAT CGC TAC GT**T TTC TCT TTT** CCT CGG ACG TAG CGA TA – 3’ |
| DNA$_{\text{hairpin}}$-12nt-loop* | 5’ – GTC TCG TAT CGC TAC GT**T TTC TCT CTT TT**C CTC GGA CGT AGC GAT A – 3’ |
| DNA$_{\text{hairpin}}$-14nt-loop* | 5’ – GTC TCG TAT CGC TAC GT**T TTC TCT CTC TTT T**CC TCG GAC GTA GCG ATA – 3’ |
| Supp. Fig. 14: circular permutation | |
| DNA$_{\text{circ_perm}}$-5’-in* | 5’ – TAC GT**T T**CC TCG GAC GTA GCG ATA **TT**G TCT CGT ATC GC – 3’ |
| DNA$_{\text{circ_perm}}$-5’-out* | 5’ – GCG ATA **TT**G TCT CGT ATC GCT ACG T**TT** CCT CGG ACG TA – 3’ |
| DNA$_{\text{circ_perm}}$-5’-in-3’-ext* | 5’ – CTA CGT **TTT T**CC TCG GAC GTA GCG ATA **TTT T**GT CTC GTA TCG **TTT CTC TCT TTT** – 3’ |

(* Overhang or loop nucleotides are underlined.)

**Table 7.** DNA oligonucleotide sequences for biotin 3'-conjugated inner guide strands in homogenous RNA-DNA hybrid nanoshapes (Fig. 6b, c, Supplementary Fig. 15, 16, 17).

| | |
|---|---|
| biotin-DNA$_{guide}$-in-13* | 5' – TTT **GTC TCG TAT CGC TAC GT**T TTC TCT CTC TCT TTT **GTC TCG TAT CGC TAC GT**T TTC TCT CTC TCT TTT **GTC TCG TAT CGC TAC GT**T TTC TCT CTC TCT **biotin –** 3' |
| biotin-DNA$_{guide}$-in-23* | 5' – **GTC TCG TAT CGC TAC GT**T TTC TCT CTC TTT **GTC TCG TAT CGC TAC GT**T TTC TCT CTC TTT **GTC TCG TAT CGC TAC GT**T TTC TCT CTC TCT CTC TCT CTC T **biotin –** 3' |

(* Hybridization sites in the DNA guide are underlined. Number indicates the liker length z.)

## Supplementary References


1. Dibrov, S. M., McLean, J., Parsons, J. & Hermann, Self-assembling RNA square. *Proc. Natl. Acad. Sci. USA* **108**, 6405-6408 (2011).

2. SantaLucia, J., Jr. A unified view of polymer, dumbbell, and oligonucleotide DNA nearest-neighbor thermodynamics. *Proc. Natl. Acad. Sci. USA* **95**, 1460-1465 (1998).

3. von Ahsen, N., Oellerich, M., Armstrong, V. W. & Schutz, E. Application of a thermodynamic nearest-neighbor model to estimate nucleic acid stability and optimize probe design: prediction of melting points of multiple mutations of apolipoprotein B-3500 and factor V with a hybridization probe genotyping assay on the LightCycler. *Clin. Chem.* **45**, 2094-2101 (1999).

4. Dibrov, S. M. *et al.* Structure of a hepatitis C virus RNA domain in complex with a translation inhibitor reveals a binding mode reminiscent of riboswitches. *Proc. Natl. Acad. Sci. USA* **109**, 5223-5228 (2012).
